# Low-grade myofibroblastic sarcoma of the mandible: a case report

**DOI:** 10.4076/1752-1947-3-8458

**Published:** 2009-08-10

**Authors:** Iwona Niedzielska, Tomasz Janic, Bartlomiej Mrowiec

**Affiliations:** 1 Department of Craniomaxillofacial Surgery, Medical University of Silesia, Katowice, Poland; 2 Private Dental Clinic Sawdent, Sosnowiec, Poland; 3 Private Dental Clinic Polmedico, Bielsko-Biala, Poland

## Abstract

**Introduction:**

Low-grade myofibroblastic sarcoma is a rare entity, which mostly develops in the soft tissues of the head and neck. Within the oral cavity lingual lesions are the most common. It tends to recur locally rather than to metastasise.

**Case presentation:**

We present a 54-year-old man with a one-year history of buccal oedema. He also had arterial hypertension and clinical examination revealed distension of the left mandibular ramus with laminar deflection in the area of the retromolar triangle.

**Conclusion:**

We present a rare intramandibular encapsulated lesion that caused diagnostic difficulties. Our diagnostic methods included immunohistochemistry and molecular investigations. We emphasise the uncommon location of this tumour type.

## Introduction

Low-grade myofibroblastic sarcoma (LGMS) represents a rare entity, which mostly develops in the soft tissues of the head and neck [1]. Within the oral cavity lingual lesions are the most common [2,3] and they tend to recur locally rather than to metastasise. Diagnostic methods included immunohistochemistry and molecular investigations.

## Case presentation

**Figure 1:**
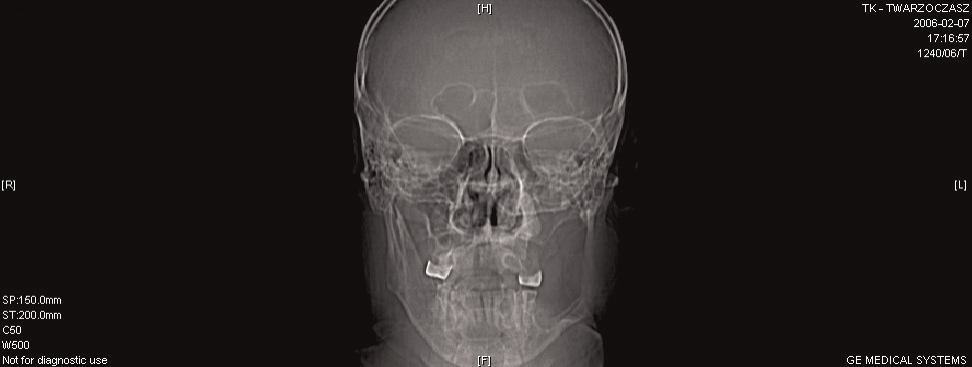
**Cranial X-ray**. The figure demonstrates a well-delineated osseous defect spreading throughout the left mandibular ramus, infiltrating and destroying the structures of the pterygopalatine fossa.

In February 2006 a 54-year-old, white, Caucasian man was seen in the Outpatient Clinic of the Craniomaxillofacial Surgery Department in Katowice with a one-year history of buccal oedema. He also had arterial hypertension. A clinical examination revealed distension of the left mandibular ramus with laminar deflection in the area of the retromolar triangle. Pantomogram and cranial X-rays demonstrated a well-delineated osseous defect spreading throughout the left mandibular ramus, infiltrating and destroying the structures of the pterygopalatine fossa (Figure 1).

**Figure 2:**
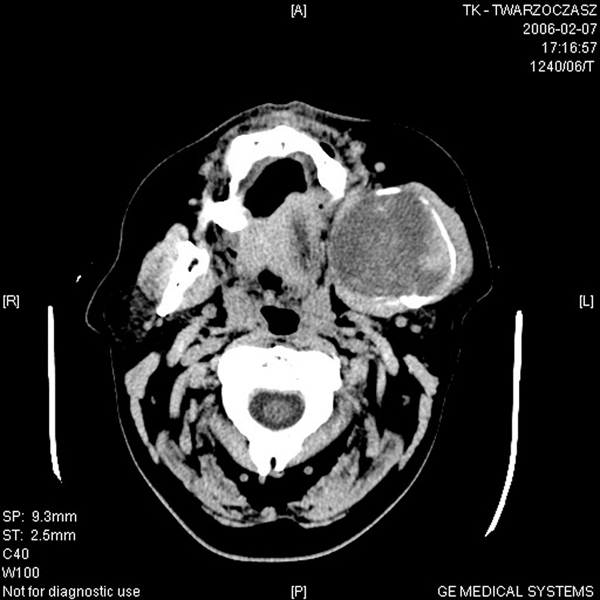
**Computed tomography scan**. The figure demonstrates a tumorous lesion in the area of the mandibular angle and ramus, slightly and heterogeneously enhanced after an intravenous contrast agent.

Fine needle aspiration biopsy (FNAB) was non-contributory. A computed tomography (CT) scan of the facial area revealed a tumorous lesion in the area of the mandibular angle and ramus 59 × 54 mm in size which was slightly and heterogeneously enhanced after an intravenous contrast agent was introduced, causing bone distension (Figure 2). The mass may possibly have infiltrated the left masseter muscle, and it adhered closely to the hard palate resulting in its deformation; invasion could also not be ruled out. It also revealed nasopharyngeal crevice deformity, invasion of the subtemporal fossa and partial thinning of the posterior maxillary sinus wall. Tissue density changes visible within the maxillary sinus were possibly consistent with mucous membrane thickening. There was no lymph node enlargement.

Tumour enucleation was performed including the periosteum and capsule. The tumour, which had a gelatinous consistency, exhibited a tendency to invade the subtemporal fossa. Clear margins were obtained and the histology result was low-grade myofibroblastic sarcoma. The mitotic index was 5f.p. × 10eHPF. At the time of writing, no recurrence or metastases have been found.

Macroscopically the tumour was approximately 5 cm in diameter, grey-white, hard and fibrous and there was no necrosis. It was removed, along with part of the bone, and microscopy demonstrated a tumour composed of spindle cells arranged in interlacing bundles. The nuclei were mostly fusiform and elongated with some round or polymorphic nuclei also present. Most tumour cells showed low grade atypia while polymorphonuclear cells showed high-grade atypia. The mitotic index was low (MI up to 5/10 HPF). The nuclei of some of the tumour cells were hyperchromatic with tiny acidophilic nucleoli and the cytoplasm was pale pink. No necrotic foci were seen. There was oedema in the central part of the tumour with pseudomyxoid and microcystic areas, and the inner and peripheral parts showed thick bundles of collagen fibres, local hyalinisation and the disappearance of tumour cells and keloid-like areas.

**Figure 3:**
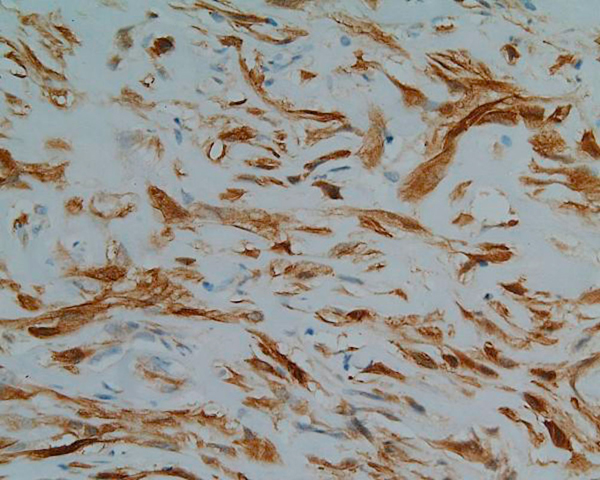
**The histopathological picture (magnification ×250)**. Strong calponin positivity of tumour cells.

**Figure 4:**
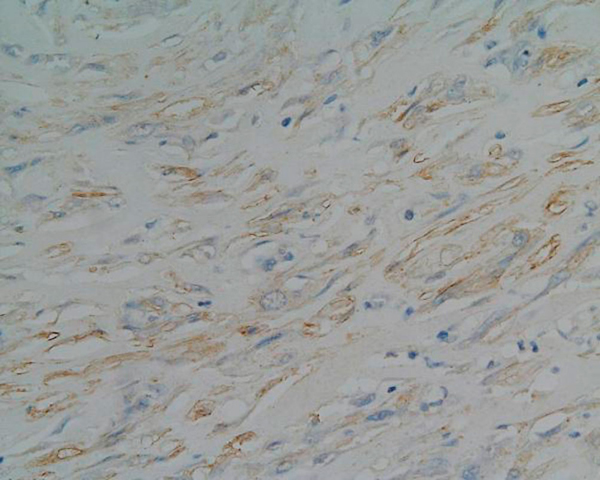
**The histopathological picture (magnification ×400)**. Focal CD99 positivity of tumour cells.

**Figure 5:**
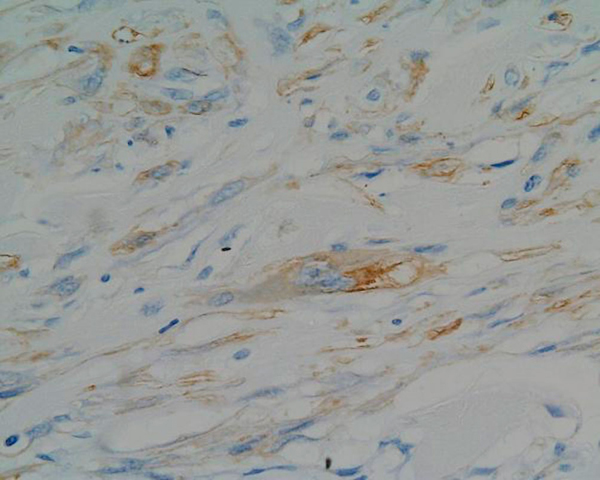
**The histopathological picture (magnification ×320)**. Focal SMA positivity of tumour cells.

Tumour blood vessels showed considerable variation from tiny, round and elongated to larger ones exhibiting wall thickening and hyalinisation. An analysis of the tumour pattern revealed mild chronic inflammation and microscopic examination also showed bone trabeculae invasion. The tissue margins were clear. At immunohistochemistry, the specimen stained positively for calponin (Figure 3), vimentin, actin, CD99 (Figure 4), and focally for SMA (Figure 5). CD34, S100 and desmin stainings were negative. Oil-red stain was also negative. The histological picture was consistent with a low-grade myofibroblastic sarcoma.

## Discussion

Although the patient's age and gender were consistent with literature reports on low-grade myofibroblastic sarcomas, we would like to emphasise the uncommon location of this tumour type (within the mandible) as well as a non-typical macroscopic appearance (the presence of a capsule). Initial histological diagnosis was inconsistent with the clinical condition. However, problems with differentiation between sarcoma subgroups have been described in the literature with the most common locations being the oral cavity (tongue) followed by the limbs, pelvis, lungs and breasts, with a predilection for soft, perifascial and subcutaneous tissues [2]-[4]. Other locations have also been described: the salivary gland [5], nasal skin [6] and the vulva [7]. Painless growth of a large mass is typical of intraosseous tumours [8]. With other locations some signs may occur such as fever, chills, leukocytosis or meningeal irritation (cerebral tumour), and more aggressive growth has been observed in the abdomen. Mentzel et al. found recurrence in two, and metastases in one, of his 18 patients [3]. Surgical resection with clear margins is the treatment of choice. However, a one-year survival was reported following a non-radical resection [2]; our case of an encapsulated tumour seems to confirm such a possibility.

## Conclusions

Radiological examinations of a low-grade myofibroblastic sarcoma of pelvic bones and limbs reveal a well-demarcated osteolytic lesion with no periosteal reaction [8]; our results were similar. Diagnostic immunocytochemistry and molecular investigations should be performed. Tumour cell positivity is characteristic for calponin, MDM-2 and PDGFRα. Diagnostic problems have been encountered when differentiating from leiomyosarcoma [9] and the prognosis depends on the malignancy grade. Guillou et al. tried to predict the likelihood metastasis of low-grade myofibroblastic sarcomas [10]. A high malignancy grade (high mitotic index), a tumour size of >10 cm and a deep location increase the tendency for metastasis.

## Abbreviations

CT: computed tomography; FNAB: fine-needle aspiration biopsy; LGMS: low-grade myofibroblastic sarcoma; MDM-2: transformed 3T3 cell double minute 2; PDGFRα: platelet-derived growth factor receptor, alpha polypeptide.

## Consent

Written informed consent was obtained from the patient for publication of this case report and any accompanying images. A copy of the written consent is available for review by the Editor-in-Chief of this journal.

## Competing interests

The authors declare that they have no competing interests.

## Authors' contributions

IN, TJ and BM were all involved in the management of the patient as well as writing the case report. All authors have read and approved the final manuscript.
